# Coupled CFD‐DEM modeling to predict how EPS affects bacterial biofilm deformation, recovery and detachment under flow conditions

**DOI:** 10.1002/bit.28146

**Published:** 2022-06-02

**Authors:** Yuqing Xia, Pahala G. Jayathilake, Bowen Li, Paolo Zuliani, David Deehan, Jennifer Longyear, Paul Stoodley, Jinju Chen

**Affiliations:** ^1^ School of Engineering Newcastle University Newcastle upon Tyne UK; ^2^ Department of Oncology University of Oxford Oxford UK; ^3^ School of Computing Newcastle University Newcastle upon Tyne UK; ^4^ The Medical School Newcastle University Newcastle upon Tyne UK; ^5^ Department of Orthopaedics Freeman Hospital Newcastle upon Tyne UK; ^6^ Marin, Protective, and Yacht Coatings, AkzoNobel Gateshead UK; ^7^ Department of Microbial Infection and Immunity and the Department of Orthopaedics The Ohio State University Columbus Ohio USA; ^8^ Department of Mechanical Engineering, National Centre for Advanced Tribology at Southampton (nCATS), National Biofilm Innovation Centre (NBIC) University of Southampton Southampton UK

**Keywords:** biofilm mechanics, biofilm modeling, computational fluid dynamics, discrete element method, extracellular polymeric substances

## Abstract

The deformation and detachment of bacterial biofilm are related to the structural and mechanical properties of the biofilm itself. Extracellular polymeric substances (EPS) play an important role on keeping the mechanical stability of biofilms. The understanding of biofilm mechanics and detachment can help to reveal biofilm survival mechanisms under fluid shear and provide insight about what flows might be needed to remove biofilm in a cleaning cycle or for a ship to remove biofilms. However, how the EPS may affect biofilm mechanics and its deformation in flow conditions remains elusive. To address this, a coupled computational fluid dynamic– discrete element method (CFD‐DEM) model was developed. The mechanisms of biofilm detachment, such as erosion and sloughing have been revealed by imposing hydrodynamic fluid flow at different velocities and loading rates. The model, which also allows adjustment of the proportion of different functional groups of microorganisms in the biofilm, enables the study of the contribution of EPS toward biofilm resistance to fluid shear stress. Furthermore, the stress–strain curves during biofilm deformation have been captured by loading and unloading fluid shear stress to study the viscoelastic properties of the biofilm. Our predicted emergent viscoelastic properties of biofilms were consistent with relevant experimental measurements.

## Graphical abstract

Deformation and detachment of biofilms with different extracellular polymeric substance volume ratios (20%, 40%, and 51%) under the same flow conditions.

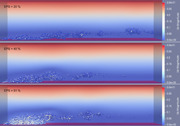

## 1 INTRODUCTION

Bacterial biofilms are initiated by reversible attachment of planktonic bacteria to a surface. Bacteria are then irreversibly attached to the surface and develop cell–cell cohesion. Matured biofilms are embedded in extracellular polymeric substances (EPS) which are produced by bacteria themselves (Flemming & Wingender, 2010). The formation of biofilm helps bacteria to survive in harsh environments such as fluid flows (Banerjee et al., 2020). It was found that bacteria in biofilms are much more resistant to antibiotics than in planktonic state (Davies, 2003). Biofilms have dramatic impacts for a wide range of industries. For example, biofilms play an important role in bioremediation since they are able to convert toxic pollutants to harmless products (Singh et al., 2006; Yadav & Sanyal, 2019). Biofilms are also essential in wastewater treatment (Capdeville & Rols, 1992; Sehar & Naz, 2016). However, the accumulation of biofilms in industrial pipelines and drinking water systems may lead to biocorrosion (Abe et al., 2012; Klapper et al., 2002). Additionally, biofilms adhered to marine surfaces is an important trigger of accelerated biofouling (Antunes et al., 2019). The biofilms attached to the ship hull increase the frictional drag resulting in higher fuel consumption (de Carvalho, 2018). The emergence of biofilms allows pathogenic bacteria to survive in diverse environments (Tasneem et al., 2018). Besides, pathogen transmission is of concern to public health and can cause infection when the cells detach from the biofilm (Brindle et al., 2011). Therefore, a greater understanding of biofilm detachment in different hydrodynamic conditions may help to control the biofilm‐related infection (Stoodley et al., 2002).

In biofilms, the EPS is a self‐produced matrix which majorly consists of polysaccharides, extracellular DNA (eDNA) and protein (Erskine et al., 2018; Gloag et al., 2013; Yadav & Sanyal, 2019). It provides many functions, such as adhesion to surfaces and cohesion to maintain the mechanical stability of the biofilm system (Flemming et al., 2007). The production of EPS is essential during biofilm development since bacteria cells could be immobilized by EPS (Flemming & Wingender, 2010). EPS production can be responsively regulated, for example, it was found that EPS production could be affected by EPS biosynthetic genes (Ali et al., 2000; Song et al., 2018). Besides, mutant strains could cause the overproduction of EPS to help the biofilm position in the beneficial environment (Hibbing et al., 2010). All these could affect the EPS amount in biofilms. Biofilms may also increase the strength of the matrix by increasing EPS production when subjected to mechanical stresses at intermediate time scales (e.g., 1 h) (Shaw et al., 2004). Different biofilms can have different EPS and different mechanical properties (Houari et al., 2008; Klapper et al., 2002; Rupp et al., 2005; Stoodley et al., 1999; Vinogradov et al., 2004; Wloka et al., 2004). However, it is difficult to quantify EPS by microscopy or chemical analysis due to the complexity of the chemistry, as well as bias in extraction and purification techniques. Although EPS is complex, computational modeling can be simplified to represent its overall physical function rather than identify the individual polymer components, hence, gaining better understanding of the contribution of EPS production to biofilm mechanical properties.

In this study, a three‐dimensional individual‐based model (IbM) of biofilm was developed by coupling the computational fluid dynamics approach (CFD) with the discrete element method (DEM). This model was implemented on NUFEB (https://github.com/nufeb) which is an open‐source tool for individual‐based modeling of microbial communities (Li et al., 2019). NUFEB integrates CFD‐DEM solver SediFoam (https://github.com/xiaoh/sediFoam) which provides a flexible interface between large‐scale atomic/molecular massively parallel simulator (LAMMPS) (Plimpton, 1995) and open‐source field operation and manipulation (OpenFOAM) (Greenshields, 2017). The framework enabled us to describe the fluid induced biofilm deformation and detachment subjected to different flow velocities. In this study, we modeled a bacterial mutant that can produce the same type of EPS at different levels. Different EPS amounts were obtained by varying the relevant kinetic parameters in the model. We predicted the effect of EPS amount on the mechanical properties of biofilms and biofilm detachment.

## 2 METHODOLOGY

**Figure 1:**
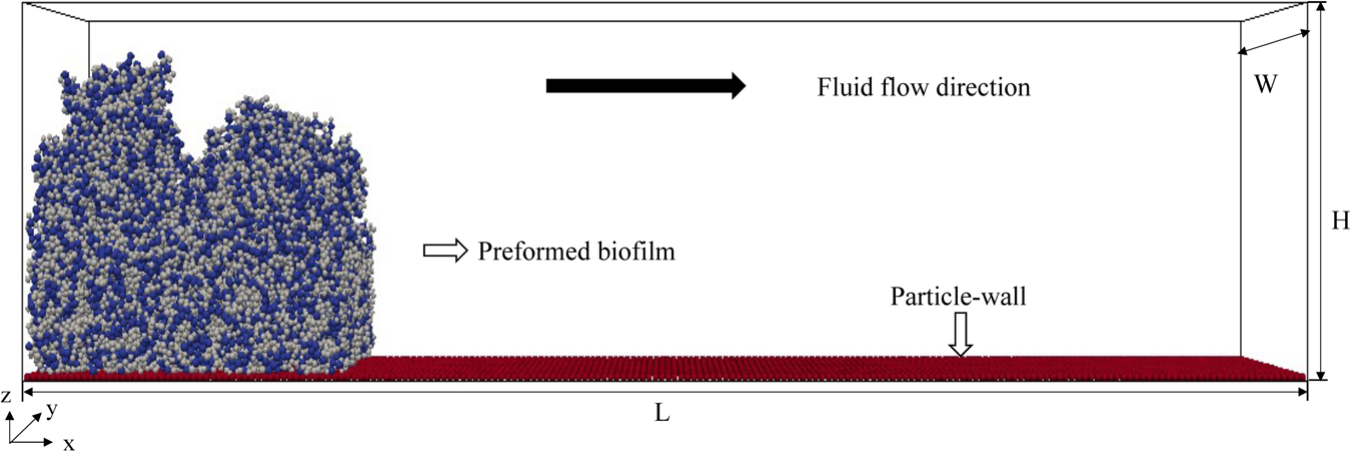
Representation of pregrown biofilm (with 51% EPS) in the channel. Bacterial cells are represented by blue particles while the gray particles are EPS agents, and red particles are a layer of the surface wall. EPS, extracellular polymeric substance.

The processes of biofilm growth and biofilm deformation were decoupled in this study, that is, fluid flow was applied to a pregrown biofilm. The pregrown biofilm was “grown” under static conditions for 5.3 days using the NUFEB individual based model which was described in Jayathilake, et al. (2017). The kinetic parameters for biofilm growth are provided in supporting information (Table S1). Only bacteria growth, division, and EPS production were considered in this study. Then the two‐way coupling between the solid biofilm and computational fluid dynamic was adopted to investigate the deformation and detachment of biofilm under different hydrodynamic conditions. The simulation domain is displayed in Figure 1, with the pregrown biofilm positioned on the inlet side of the channel. The diameter of the involved particles is in the micrometry range (0.7–1.4 μm) based on the stochasticity of the biological system (Jayathilake, et al., 2017). The fluid flow was applied along the top wall while the left and right wall have the cyclic boundary conditions (channel dimensions [L × W × H]: 200 × 30 × 50 μm^3^). Cyclic boundary conditions were also applied to the front and back walls to reduce computational effort. A no‐slip boundary condition was adopted on the bottom wall where the fluid velocity is zero. The details of cohesion among all the functional groups are discussed in Section 2.6.

### 2.1 Fluid‐induced biofilm deformation and detachment

Experimental work has shown that EPS production in biofilms varied with bacterial strains and growth conditions (Costa et al., 2018; Danese et al., 2000). In this study, we achieved different EPS volume ratio (i.e., EPS volume divided by the volume of the biofilm) by changing the EPS growth yield coefficient in the modeling. The EPS growth yield coefficient was varied from 0.12 to 0.22 (g COD_EPS_/g COD_S_) which corresponds to EPS/biofilm ratio of 20%–51% here. To investigate the biofilm deformation and detachment events, the biofilm with 46% EPS was subjected to inlet flow velocity between 0.1 and 0.4 m/s (Reynold number from 3.75 to 15, maximum wall shear stress from 10.7 to 42.7 Pa, shear rate from 2000 to 8000 s^−1^) for a duration of 40 ms. In the next simulation, the inlet fluid velocity was kept at 0.3 m/s to study the effect of EPS production on biofilm deformation and detachment. The detachment rate coefficient, which is defined as the ratio of the volume of detached biofilm clusters to the total volume of preformed biofilm, was calculated during the initial 14 ms (before biofilm washed away from the surface wall). Cluster detachment from the biofilm was defined as erosion if the particle number of the cluster was less than 1000 and sloughing if the particle number of the detached cluster exceeded 1000. The EPS amount, the mean and maximum heights, the roughness and porosity of different biofilms are summarized in Supporting Information (Table S2).

### 2.2 Biofilm deformation‐recovery test

The responses of biofilm to a rapid fluctuating shear stress were analysed immediately before biofilm failure. To save computational time, the fluid shear stress was applied to the biofilm for 3 ms (loading cycle) and then stopped immediately. Afterward, the biofilm was allowed to relax for 17 ms (unloading cycle). During the loading period, the fluid shear stress was increased by increasing the fluid velocity from 0 m/s at a constant acceleration. For the biofilm with 46% EPS, deformation‐recovery tests were carried out by exposing the biofilm to the ramping flow with different accelerations: 20, 30, and 40 m/s^2^, respectively. Then the biofilms with 40% and 51% EPS were subject to the increasing fluid velocity at the acceleration of 40 m/s^2^ to investigate the effect of EPS amount on mechanical response of biofilm. The shear strain in this simple shear test was defined as the angle change between the front edge of biofilm with the left channel wall (Figure S1). The shear modulus was calculated as follows:

(1) $$G=\frac{\sigma_{\mathrm{xz}}}{\alpha },$$

where α is the shear strain, $\sigma_{\text{xz}}$ is the fluid induced shear stress on the biofilm which was computed globally by LAMMPS (Thompson et al., 2009). In this section, three planes (*y* = 5 μm, *y* = 15 μm, and *y* = 25 μm) were selected to measure the deformation angle thus to obtain the averaged shear strain (Figure S2).

### 2.3 Motion of bacterial and EPS agents

During biofilm deformation and detachment, the motion of bacterial cells and EPS agents are tracked by DEM on a Lagrangian framework:

(2) $$m_{i}\frac{\mathrm{d}{\vec{v}}_{i}}{\mathrm{d}t}={\vec{f}}_{c,i}+{\vec{f}}_{\mathrm{coh},i}+{\vec{f}}_{\mathrm{fp},i},$$

where ${\vec{v}}_{i}$ is the velocity of the particle *i*; $m_{i}$ is the particle mass; ${\vec{f}}_{c,i}$ is the contact force among collided particles (Xia et al., 2021), ${\vec{f}}_{\mathrm{coh},i}$ is interparticle cohesive force, ${\vec{f}}_{\mathrm{fp},i}$ is the fluid‐particles interaction force.

### 2.4 Locally averaged Navier–Stokes equations for fluids

The fluid flow is solved by locally averaged incompressible Navier–Stokes equation in which the fluid density $\rho_{f}$ is constant:

(3) $$\nabla ∙\left(\epsilon_{s}{\vec{U}}_{s}+\epsilon_{f}{\vec{U}}_{f}\right)=0,$$

(4) $$\frac{\partial (\epsilon_{f}{\vec{U}}_{f})}{\partial t}+\nabla ∙\left(\epsilon_{f}{\vec{U}}_{f}{\vec{U}}_{f}\right)=\frac{1}{\rho_{f}}(-\nabla p+\epsilon_{f}\nabla ∙\vec{R}+{\vec{F}}_{\mathrm{fp}}),$$

$\epsilon_{s}$ is solid volume fraction while $\epsilon_{f}$ is fluid volume fraction which equals to (1 − $\epsilon_{s}$). ${\vec{U}}_{s}$ and ${\vec{U}}_{f}$are particle velocity and fluid velocity, respectively. ${\vec{F}}_{\mathrm{fp}}$ is the fluid–particle interaction force. ∇p is the pressure gradient, $\vec{R}$ is the stress tensor consisting of viscous stress and Reynolds stress, only viscous stress was computed since the Reynolds number is small here (3.75–15). The Eulerian field $\epsilon_{s}$, ${\vec{U}}_{s}$, and ${\vec{F}}_{\mathrm{fp}}$ are calculated by averaging the Lagrangian information of particles (Sun et al., 2018).

### 2.5 Fluid–particle interaction

In this model, the fluid–particle interaction force ${\vec{f}}_{\mathrm{fp},i}$ consists of a drag force and lift force. For the particle i, the drag force model is expressed as (Sun et al., 2018):

(5) $${\vec{f}}_{\mathrm{fp},i}^{\mathrm{drag}}=\frac{V_{p,i}}{\epsilon_{f,i}\epsilon_{s,i}}\beta_{i}({\vec{U}}_{f,i}-{\vec{u}}_{p,i}),$$

where $V_{p,i}$ is the volume of the particle *i*, ${\vec{U}}_{f,i}$, and ${\vec{u}}_{p,i}$ are the fluid velocity and particle velocity, respectively. $\epsilon_{f,i}$ is the fluid volume fraction while $\epsilon_{s,i}$ is the solid volume fraction, $\beta_{i}$ is the drag correlation coefficient which is used to convert terminal velocity correlation to drag correlation (Syamlal et al., 1993).

In addition, the lift force on the particle i is calculated by the following formula (Sun et al., 2018; Van Rijn, 1984; Zhu et al., 2007):

(6) $${\vec{f}}_{\mathrm{fp},i}^{\mathrm{lift}}=C_{l}{\left(\rho_{f}\mu \right)}^{0.5}d_{p,i}^{2}\left({\vec{U}}_{f,i}-{\vec{u}}_{p,i}\right)\times \frac{\omega_{i}}{{\left|\omega_{i}\right|}^{0.5}},$$

where $C_{l}$ is the lift coefficient equals to 1.6, $\omega_{i}=\nabla \times {\vec{U}}_{f,i}$ is the curl of the flow velocity interpolated to the center of particle i.

### 2.6 Cohesive force among particles

The cohesive force among the particles was computed by using the equation below (Israelachvili, 2011; Sun et al., 2018):

(7) $${\vec{F}}_{\mathrm{coh},i}=-\frac{A}{6}\frac{64r_{i}^{3}r_{j}^{3}(s+r_{i}+r_{j})}{{\left(s^{2}+2r_{i}s+2r_{j}s\right)}^{2}{\left(s^{2}+2r_{i}s+2r_{j}s+4r_{i}r_{j}\right)}^{2}}{\vec{n}}_{\mathrm{ij}}$$

where *A* is the cohesive strength, and *s* is the separation distance between the particle surface. A minimum separation distance $s_{\min}$ was implemented when the separation distance between the two particles equals zero (s=0). In this study, five different values of cohesive strength were used for the interactions of bacterial cells with bacterial cells, bacteria cells with EPS agents, bacteria cells with particle‐wall, EPS agents with the particle‐wall, EPS agents with EPS agents. Since EPS plays a significant role on binding the bacterial cells, the cohesive strength driven by EPS was assumed to be three orders of magnitude larger than that for bacteria (Fang et al., 2000). The mechanical parameters of the simulations are listed in Table 1. The fluid density was 10^3 ^kg m^−3^ and the fluid dynamic viscosity was 10^−3 ^kg m^−1^ s^−1^. The contact model for the colloid particles has been introduced in Xia et al. (2021). Throughout the manuscript and supporting information, the results were presented as averaged values with standard deviations.

**Table 1:** The mechanical and physical parameters of the biofilm used in our simulations

Numerical simulation parameters		
Density of particles	10^3 ^kg m^−3^	Xia et al. (2021)
Normal and tangential elastic constants	10^3 ^kg m^−1^ s^−2^	Böl et al. (2013)
Normal damping constants	10^13 ^m^−1^ s^−1^	Chosen
Tangential damping constants	10 m^−1^ s^−1^	Chosen
Parameters for cohesive model		
Particle interaction	Cohesive strength	
Bacteria–EPS	1.6 × 10^−18 ^J	Chosen
Bacteria–particle wall	2.3 × 10^−21 ^J	Lower (2005)
EPS–particle wall	2.3 × 10^‐18 ^J	Chosen
EPS–EPS	5 × 10^−18 ^J	Fang et al. (2000)
Bacteria–HET bacteria	1.6 × 10^−21 ^J	Bos et al. (1999)

Abbreviation: EPS, extracellular polymeric substance.

## 3 RESULTS AND DISCUSSION

### 3.1 Flow effect on biofilm deformation and detachment

**Figure 2:**
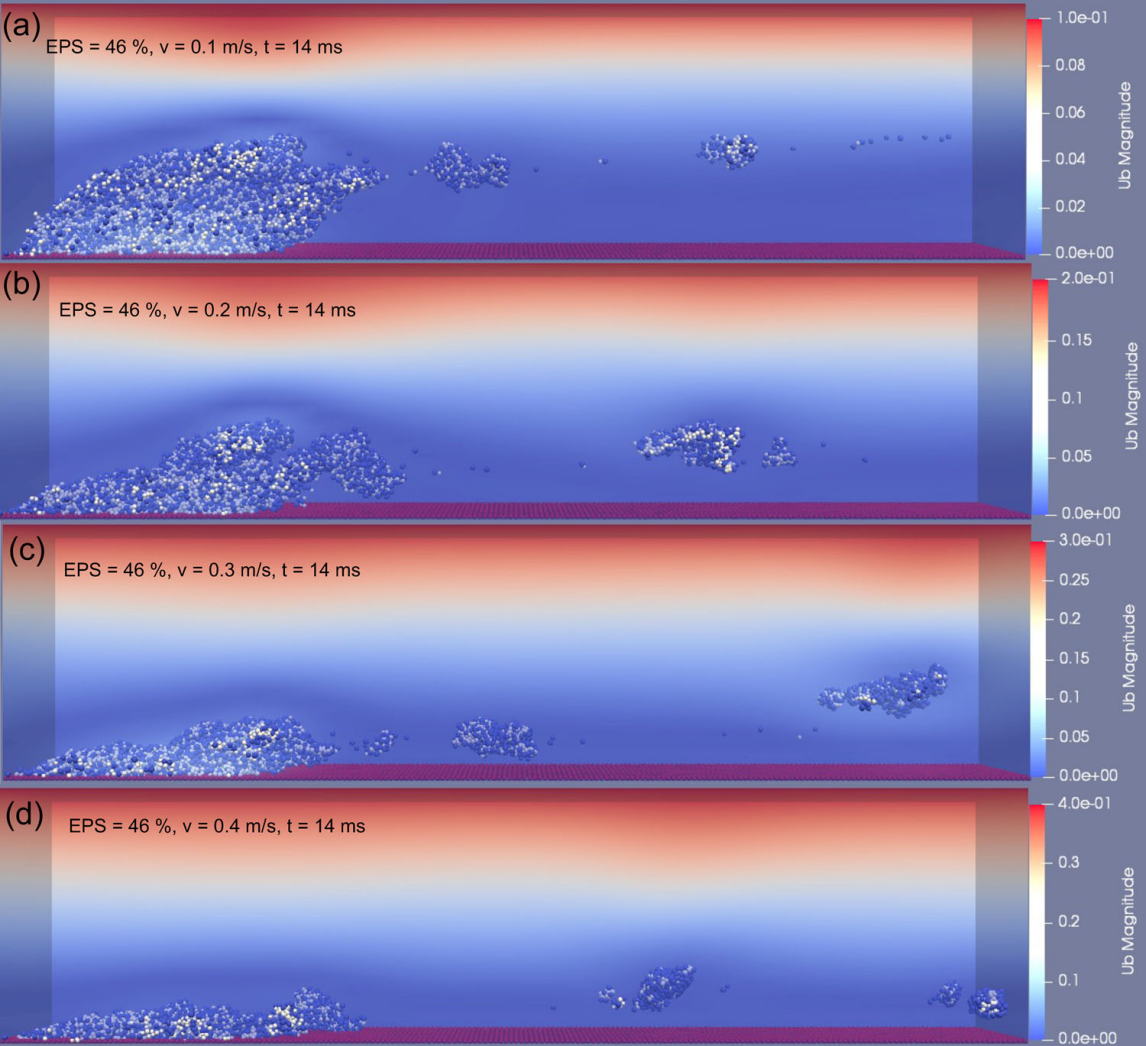
Biofilm (with 46% extracellular polymeric substance) deformation and detachment at inlet flow velocity in the range of (a) 0.1 m/s, (b) 0.2 m/s, (c) 0.3 m/s, (d) 0.4 m/s, *t* = 14 ms.

**Figure 3:**
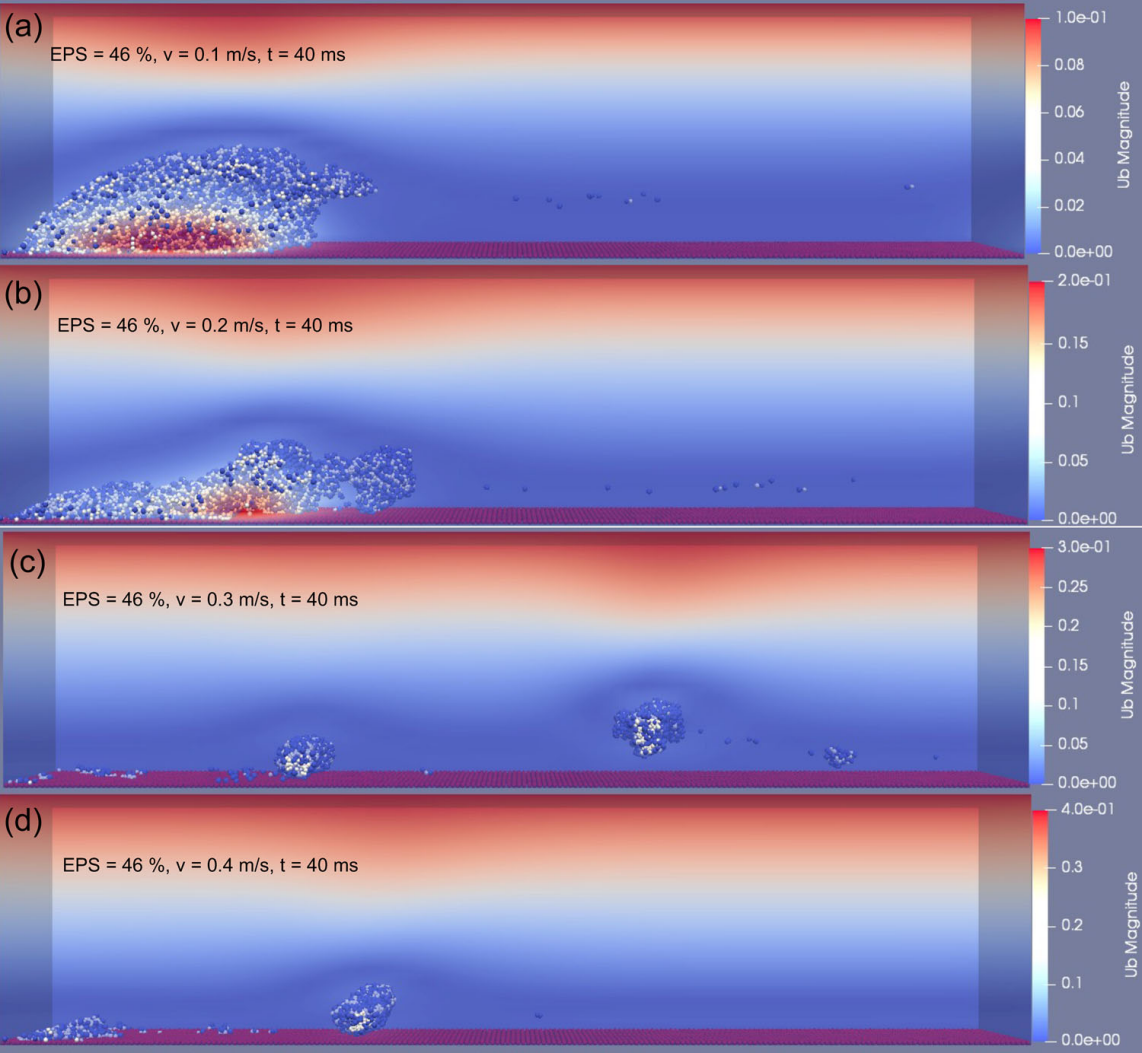
Biofilm (with 46% extracellular polymeric substance) deformation and detachment at inlet flow velocity in the range of (a) 0.1 m/s, (b) 0.2 m/s, (c) 0.3 m/s, and (d) 0.4 m/s, *t* = 40 ms.

In this study, the time was very short during the flow tests, therefore, bacterial growth and EPS production were negligible. Deformation and detachment of the biofilm with 46% EPS at four inlet flow velocities are shown in Figures 2 and 3, which are representations of the biofilm at 14 and 40 ms time points, respectively. At the lowest inlet flow velocity of 0.1 m/s, the biofilm elongated along with the flow direction and detachment occurred at the rear part of the biofilm (Figure 2a). Because of the gradient fluid shear force along the *z* direction and the patchy structure of the biofilm, the top of the biofilm deformed much more than its bottom. Biofilm deformation was dominant during exposure to this lowest level fluid shear force and only erosion occurred. No further erosion or detachment were observed between 14 and 40 ms (Figure 3a).

When the inlet flow velocity was increased to 0.2 m/s, detachment at the rear part of the biofilm occurred as early as 3 ms (Figure S3). Compared to the lowest inlet fluid velocity (i.e., 0.1 m/s), detachment frequency increased sharply, both biofilm erosion and sloughing took place (Figure 2b). As expected, the comparison of Figure 2a,b illustrates the higher flow velocity led to an increase in detached biofilm volume at the same duration. However, the biofilm was not removed at the end of simulation when the inlet flow velocity is 0.2 m/s (Figure 3b).

High‐frequent biofilm detachment events could also be captured by further increasing the inlet flow velocity to 0.3 m/s. At this higher flow, biofilm sloughing was the predominant behavior during the detachment process. A small fraction of biofilm still adhered to the surface at 14 ms (Figure 2c). The remainder biofilm, which in continuous exposure to the fluid shear force, experienced more biofilm cluster detachment and only a layer of biofilm was left at 25 ms (Figure S4A). Interestingly, this remaining biofilm layer started rolling along the rough wall under the steady fluid shear force after then (Figure S4B‐D). Finally, biofilm moved out of the original location (the initial surface occupied by the pregrown biofilm) by this rolling motion at around 40 ms (Figure 3c). This phenomenon was firstly observed by Rupp et al. in their experiments, in which *Staphylococcus aureus* (a common biofilm forming pathogen associated with medical implants) microcolonies moved downstream by rolling in a flow cell (Rupp et al., 2005).

At the highest inlet fluid velocity, 0.4 m/s, biofilm clusters with different sizes detached rapidly due to the high fluid shear force. Figure 2d displays the morphology of the biofilm after being subjected to the shear force for 14 ms, only a thin layer of biofilm remained adhered to the surface. Furthermore, the biofilm rolling motion was also captured at this flow speed. The biofilm rolled along the surface for several microseconds then lifted from the surface by the fluid (Figure S5A‐D). Eventually, the biofilm was washed away along the direction of the fluid flow (Figure 3d).

### 3.2 EPS effect on biofilm deformation and detachment

To study how the EPS amount affected the deformation and detachment of biofilms, we examined a single fluid flow condition, 0.3 m/s sustained for a duration of 40 ms. For biofilms with a low amount of EPS (20% EPS), the biofilm clusters could easily detach from the biofilm matrix (erosion‐dominated) at high frequency, accompanied by the escape of single bacterial cells (Figure S6). This may be due to the limited EPS availability to immobilize the cells in biofilm (Flemming & Wingender, 2010).

**Figure 4:**
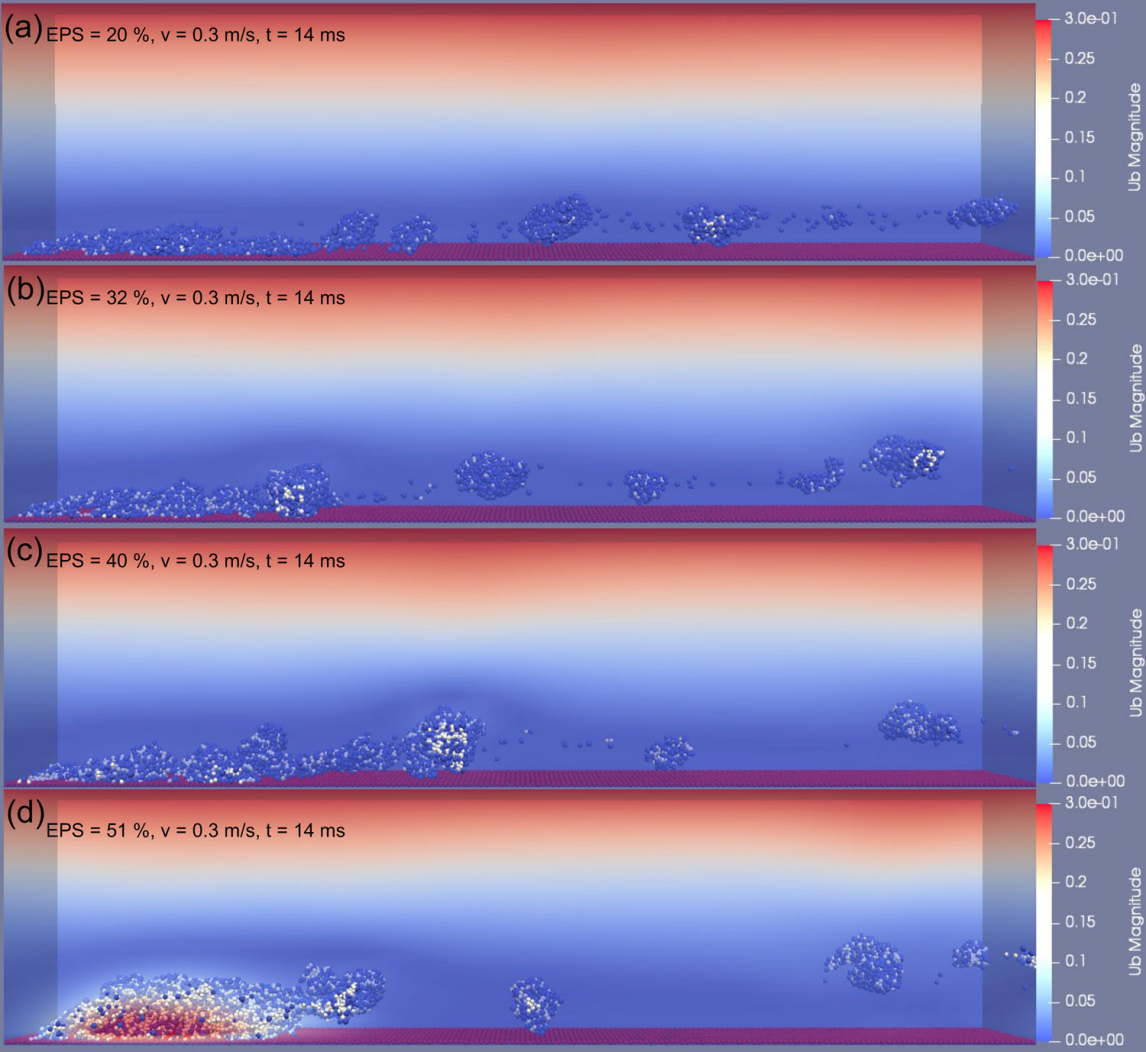
Biofilm deformation and detachment at time of 14 ms with the inlet fluid velocity of 0.3 m/s. The volume ratio of extracellular polymeric substance within the biofilm increased from (a) 20%, (b) 32%, (c) 40% to (d) 51%.

**Figure 5:**
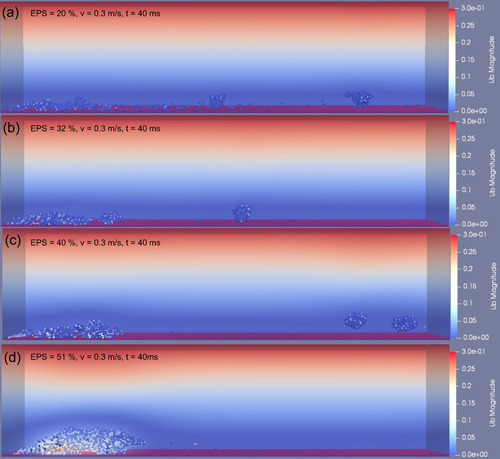
Biofilm deformation and detachment at time of 40 ms with the inlet fluid velocity of 0.3 m/s. The volume ratio of extracellular polymeric substance within the biofilm increased from (a) 20%, (b) 32%, (c) 40% to (d) 51%.

The detachment frequency of biofilm decreased with the increase in EPS amount. Figure 4 shows the biofilms after being subjected to the fluid flow for 14 ms. The volume of detached biofilm decreased as the EPS amount increased. For the biofilm with low EPS amount (20%, 32%, and 40% EPS), most biofilm were detached but a thin layer remained adhered to the surface wall at the end of 40 ms (Figure 5a‐c). However, when the EPS volume ratio was increased to 46%, the biofilm was removed by a rolling motion. The results suggest that the rolling motion depends on the amount of EPS. When the EPS volume ratio further increased to 51%, about half of the initial biofilm remained on the surface at 40 ms (Figure 5d). It is evident that the same flow caused less detachments for biofilm with higher EPS amount, which further suggests that the biofilm with greater amounts of EPS was stiffer and resisted well against fluid flows.

**Figure 6:**
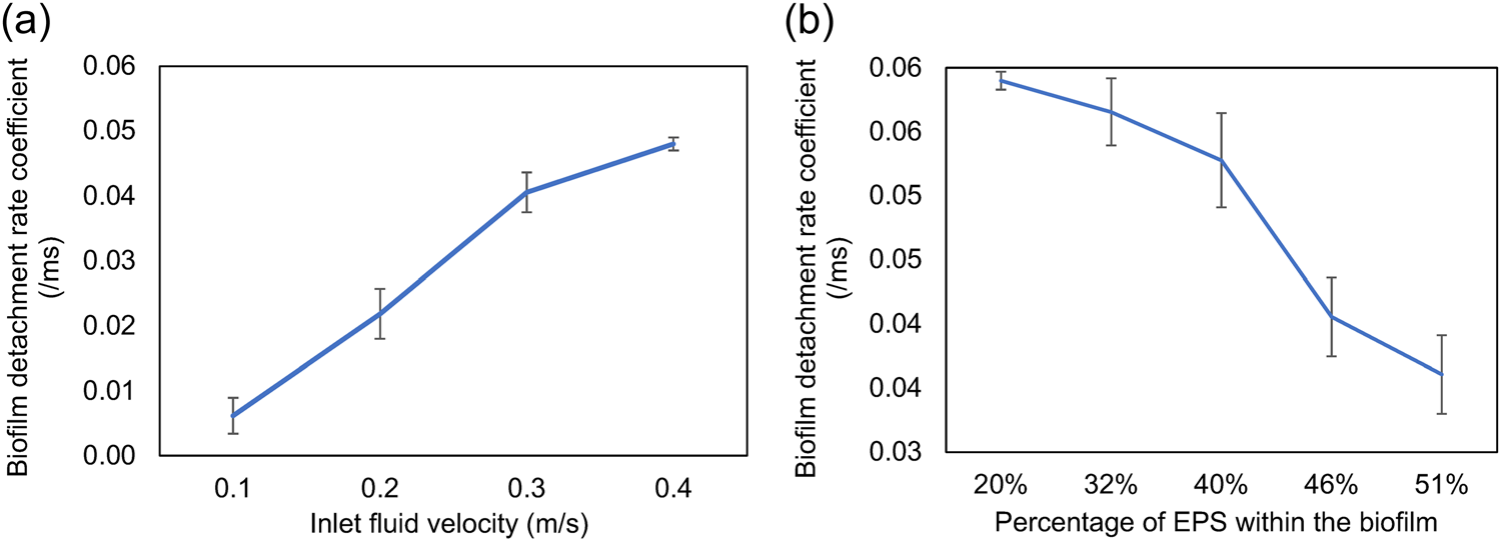
(a) The effect of fluid velocity on biofilm detachment rate coefficient for a typical biofilm with 46% EPS. (b) The effect of EPS amount on biofilm detachment rate coefficient for a given inlet flow velocity of 0.3 m/s. The error bars represent standard deviations based on three replicates. EPS, extracellular polymeric substances.

The local detachment, such as erosion and sloughing, could be significantly captured within the time of 14 ms. However, biofilm removal occurred after this period when the inlet fluid velocity was greater than 0.2 m/s. Therefore, the detachment rate coefficient, which is defined as the ratio of the volume of detached biofilm cluster to the total volume of biofilm per millisecond, was calculated during the initial detachment period (14 ms) and adopted to describe the biofilms detachment behavior under the range of hydrodynamic conditions. Each simulation was run for three replicates and the average results were calculated. As displayed in Figure 6a, the detachment rate coefficient increased with the inlet fluid velocity which agrees with previously reported experimental observations (Stoodley et al., 2002). There was no significant detachment until inlet flow velocity increased to 0.2 m/s, the coefficient increased sharply before the inlet flow velocity reached 0.3 m/s and then slowed down when the inlet fluid velocity was further increased. In keeping with the visual results (Figure 4), a negative correlation was found between the EPS amount and detachment rate coefficient when the inlet fluid velocity was kept constant (Figure 6b). The detachment rate coefficient for the biofilm with 20% EPS was approximately twice that for the biofilm with 51% EPS. The results suggest that the resistance of the biofilm to the external fluid is largely attributable to the EPS amount. EPS is responsible for the mechanical stability of the biofilm due to its cohesive properties, therefore, the biofilms with a greater density of EPS components are predicted to be more stable when exposed to the fluid flow.

### 3.3 Biofilm viscoelastic response during deformation‐recovery test

**Figure 7:**
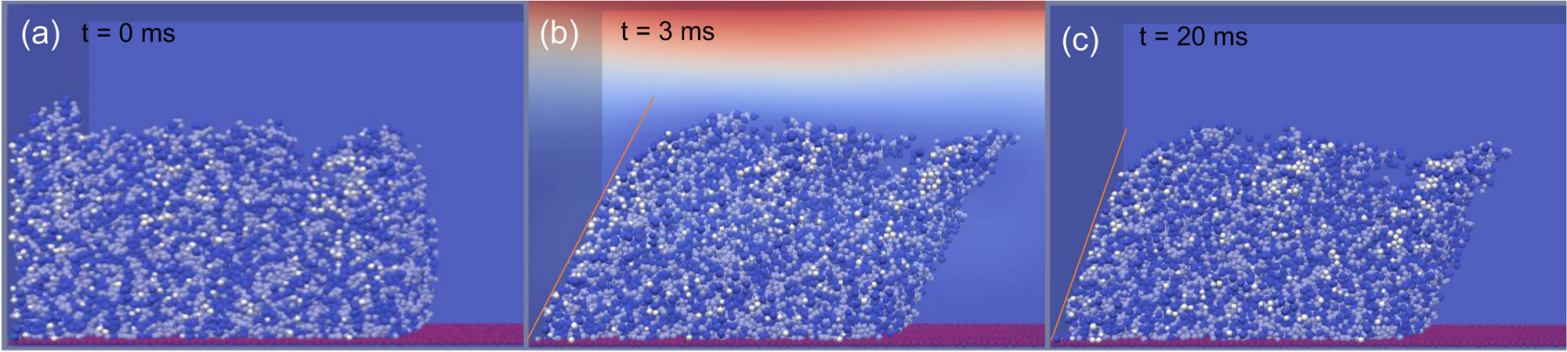
(a) The original shape of a biofilm with 46% extracellular polymeric substance, (b) maximum biofilm deformation in flow, and (c) biofilm relaxed for 17 ms after fluid flow was stopped.

Deformation of the biofilm with 46% EPS was monitored for 3 ms as the fluid velocity was incrementally increased from 0 m/s at a constant acceleration. Then the fluid flow was stopped, the biofilm was allowed to relax for 17 ms. The stress–strain curve was obtained from the loading and unloading cycle. Figure 7 shows the deformation and recovery properties of the biofilm (46% EPS). In this case, the fluid velocity was accelerated at 20 m/s^2^ and reached the maximum value (0.06 m/s) at 3 ms. The maximal deformation angle was captured at the same time (Figure 7b), approximately 25.3 degree (0.44 rads). After the fluid flow was stopped, the biofilm started to recovery. As seen in Figure 7c, the biofilm had not returned to the original shape after the full relaxation time, about six times of the duration of flow induced biofilm deformation. The results were not surprising for our simulation, as the interactions between the biofilm particles were modeled as spring‐dashpot based viscoelastic models. It matches observations of real world biofilms, as this kind of residual strain was also be observed in Klapper et al. (2002), and such a residual deformation is due to the viscous nature of biofilm (Jafari et al., 2018).

**Figure 8:**
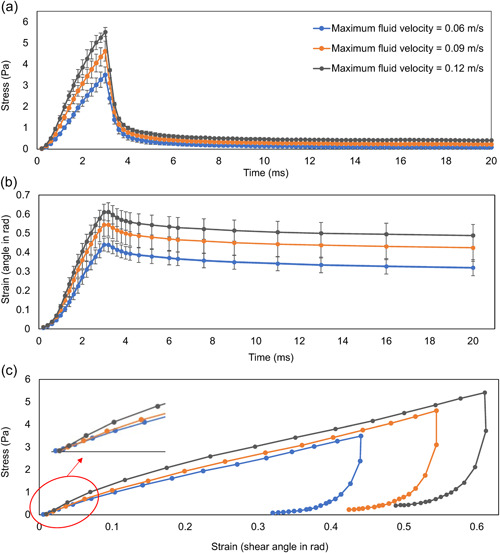
The fluid induced (a) stress and (b) strain on biofilms changed with time and the corresponding averaged (c) stress–strain curve (standard deviation did not give here for the high resolution). Where the flow was applied on the biofilm with 46% extracellular polymeric substance for 3 ms, accelerated at 20, 30, and 40 m/s^2^, to reach the peak velocities of 0.06, 0.09, and 0.12 m/s. The error bars represent standard deviations based on three replicates.

To understand the viscoelastic deformation of biofilms at different loading rates and stresses, additional simulations were performed by accelerating the fluid at 30 and 40 m/s^2^ for 3 ms, with the corresponding peak fluid velocities of 0.09 and 0.12 m/s, respectively. Figure 8a shows the fluid‐induced shear stress on biofilms overtime. It is evident that the higher flow acceleration resulted in higher shear stress imposed on biofilms (Figure 8a). This can lead to larger deformation (or shear strain) and deformation rate of biofilms (or strain rate), as seen in Figure 8b.

After the flow was removed at 3 ms, the fluid induced stress in the biofilm decreased rapidly (3–4 ms, Figure 8a), and some of the deformation (8%–10%) was immediately recovered attributable to a time‐independent elastic response. Afterward (4–20 ms, Figure 8a), the stress decay slowed down dramatically and almost reached a plateau at the end of the recovery. A residual deformation (or strain) during biofilm relaxation was captured in each deformation – recovery test and increased with the maximum fluid velocity. Such a strain rate‐dependent recovery was due to the nature of viscoelastic models adopted within the biofilms and is a common characteristic for viscoelastic materials (Capurro & Barberis, 2014; Chen et al., 2011).

Figure 8c shows the corresponding stress–strain curve of biofilms during biofilm deformation (0–3 ms) and recovery process (3–20 ms). At the lowest shear stress and shear rate, the stress could recover to zero within the allotted 17 ms of relaxation time while a full recovery could not be achieved for higher shear stresses with higher strain rates. The hysteresis loop in the curve represents the dissipation of energy during the biofilm deformation process. The area of hysteresis loop increased with the maximum fluid velocity, which suggested more energy dissipation. The calculated apparent shear moduli of biofilms (Equation 1) determined at small deformation (strain < 0.1), were 10.75 ± 1.28, 12.41 ± 1.24, and 15.21 ± 1.94 Pa at given fluid velocities (0.06, 0.09, and 0.12 m/s), respectively. The apparent shear modulus is affected by the deformation rate (or strain rate) due to the nature of viscoelastic effect. It is expected that the lower deformation rate leads to a smaller apparent shear modulus, which is also seen in this study. If we take into account different deformation rates of biofilms at different fluid velocities, it yields consistent equilibrium shear modulus and viscosity which are in the range of 3.8–4.8 Pa and 6.9–8.7 mPa*s, when using the Prony series viscoelastic model for curve fitting at different deformation rates (Chen & Lu, 2012).

To study the EPS effect on biofilm mechanics, we also focused on biofilms containing higher EPS amounts (40%, 46%, and 51%) subjected to the ramping fluid velocity at a constant acceleration of 40 m/s^2^. Biofilms with lowers EPS amount were not considered here since they could easily detach, thus the stress–strain curve would not be captured during deformation.

**Figure 9:**
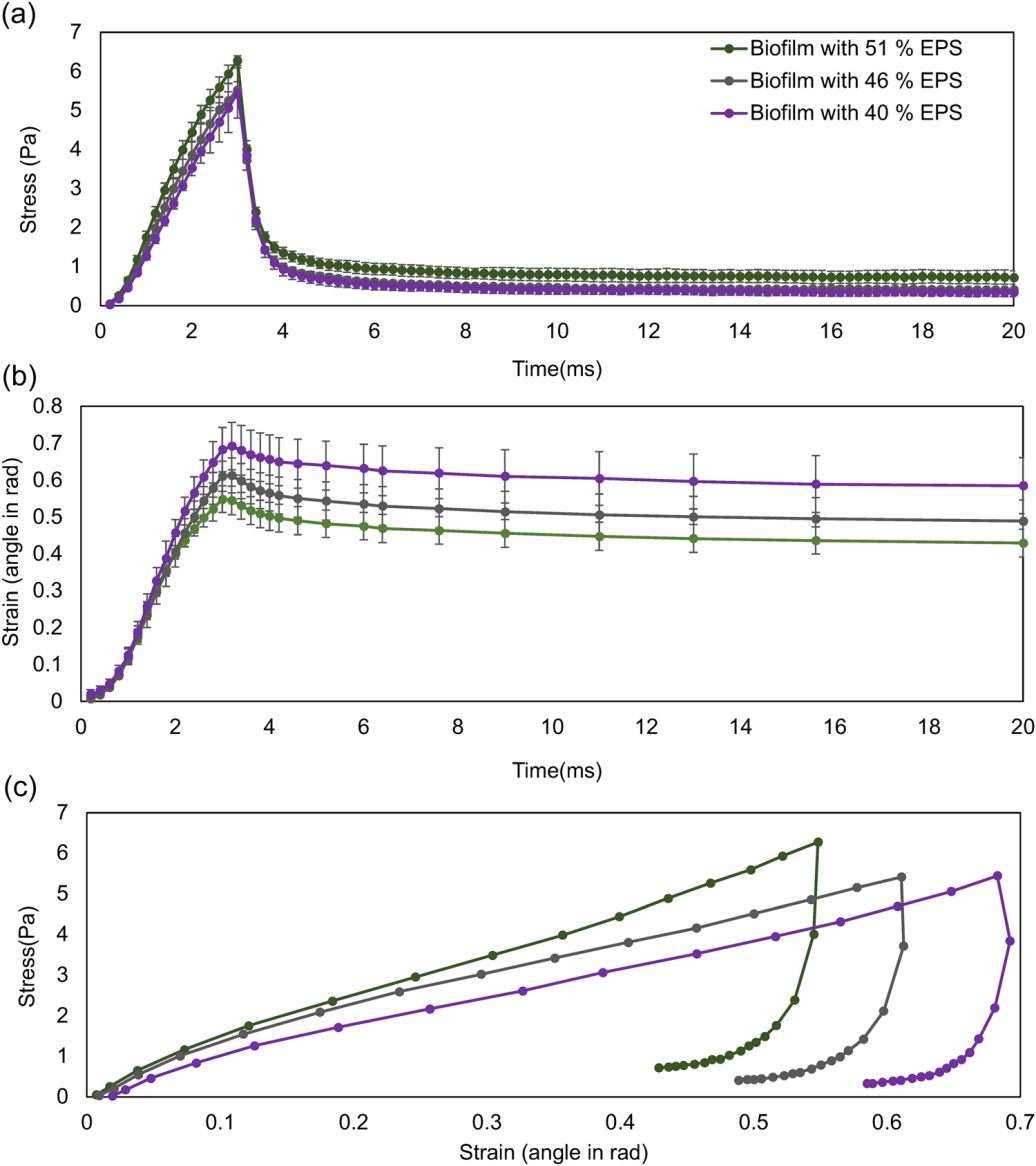
The fluid induced (a) stress and (b) strain on biofilms changed with time and the corresponding averaged (c) stress–strain curve when the flow was applied and terminated (standard deviation did not give here for the high resolution). The biofilms with 40%, 46%, and 51% extracellular polymeric substance were selected. The fluid velocity was applied at an acceleration of 40 m/s^2^. The error bars represent standard deviations based on three replicates.

Figure 9a,b show the fluid induced stress and strain changes overtime. For biofilms with 40% and 46% EPS, the fluid induced stresses on the biofilm were similar, but the peak shear stress on the 51% EPS biofilm (Figure 9a) was higher. The different stress profiles could be attributed to the different height profiles of the biofilms. Since the fluid flow was applied along the top wall in the simulation domain, the velocity varied with the height of simulation box (Figure S7). Therefore, it is important to note that although the inlet fluid condition was set as the same, the biofilms would be subjected to different fluid shear force if their height varied due to growth.

The deformation of the biofilm with 40% EPS was greater compared to biofilm with 46% EPS, and both experienced similar shear stresses. This suggests that higher EPS resulted in the better resistance to the external fluid shear force. However, the shear strain of the 51% EPS biofilm was lowest although the maximum stress was almost 16% higher than for its counterpart biofilms. Taken together, the results suggest that biofilms with higher EPS might be stiffer, which agrees with what was found in Gloag et al. (2020). As seen in Figure 9c, the stress–strain curve was almost linear at very small strains (<0.1), which was also found in our experimental measurements for flow induced biofilm deformation of *Bacillus subtilis* (Figure S8). When ignoring the deformation rate (or strain rate) effect, the apparent shear modulus at given loading conditions was 12.82 ± 2.03, 15.21 ± 1.94, and 17.18 ± 3.3 Pa for biofilms with 40% EPS, 46% EPS, and 51% EPS, respectively. When the deformation rates were accounted for, it yields the equilibrium shear modulus of 3.5 ± 0.7 Pa, 5.7 ± 1.7 Pa, and 5.6 ± 0.4 Pa for those three biofilms when using the Prony series viscoelastic model for curve fitting at different deformation rates (Chen & Lu, 2012). These are consistent with several biofilms such as *Pseudomonas aeruginosa* (Stoodley et al., 1999) and *Staphylococcus aureus (*Rupp et al., 2005). The reported corresponding viscosity for those three biofilms is 8.4 ± 4.5, 6.6 ± 1.6, 8.3 ± 3.2 mPa*s, respectively. There is no evidence for correlation between the equilibrium shear modulus and viscosity of biofilms, as found in other recent studies (Houari et al., 2008; Klapper et al., 2002; Rupp et al., 2005; Safari et al., 2015; Stoodley et al., 1999; Vinogradov et al., 2004; Wloka et al., 2004). The predicted viscosity was the resultant of the mechanical interactions for bacteria–bacteria, bacteria–EPS and EPS–EPS.

In general, all the simulated biofilms exhibited some strain stiffening effect followed by strain softening at larger strains, which is due to the viscoelastic properties and the change in the microstructure of biofilms during the deformation (Figure 9c). This is consistent with experimental measurements of biofilms at a wide range of strains (Jana et al., 2020). ANOVA test was used for statistical analysis (*α* = 0.05). The result (*p* < 0.05) suggests that the change of shear modulus with EPS amount has a statistically significant difference.

After the fluid was stopped, the stress on the biofilms decayed exponentially and the deformation recovered slowly which is a common feature for viscoelastic materials (Chen & Lu, 2012). The overall biofilm deformation recovery was 16%, 20%, and 22% for biofilms with 40%, 46%, and 51% EPS within the simulation period, respectively. The results suggest that the abundant presence of EPS in the deformed biofilms makes a significant contribution to their mechanical recovery, as the bacterial cells are much more loosely associated with other bacterial cells than EPS agents. Similar results were also verified in the experimental work, which released that the EPS is required to induce bacterial rearrangement during stress relaxation (Peterson et al., 2014).

## 4 CONCLUSIONS

A CFD‐DEM coupled model developed here has enabled us to predict biofilm deformation and detachment under varied hydrodynamic conditions. When the biofilm was exposed to a steady fluid shear force (inlet fluid velocity was kept as constant), the detachment rate increased with inlet fluid velocity (i.e., shear stress). When the inlet flow velocity was below 0.1 m/s, the biofilm deformed along the fluid direction with sparse erosion. Biofilm sloughing occurred when the inlet flow velocity increased to 0.2 m/s. When the inlet fluid velocity reached 0.3 and 0.4 m/s, the detachment events were dominated by sloughing and the remainder biofilm layer was removed in a rolling motion.

At a given inlet fluid velocity of 0.3 m/s, the detachment rate coefficient decreased with EPS amount. For the biofilm with low proportional EPS (less than 32%), the biofilm easily detached from the surface and dispersion of individual cells was observed. In these cases, the limited amount of EPS was incapable of protecting the biofilm bacteria from shear stress. Biofilm detachment frequency decreased with the increase of EPS amount. The biofilms were stiffer at higher loading rate, which is a typical characteristic for viscoelastic materials. Such viscoelastic features of biofilms also led to the hysteresis loop (energy dissipation), which was predicted by the stress–strain curves in our simulations and experimental measurements (Rupp et al., 2005). The predicted shape of stress–strain curve during the flow induced deformation is similar to our measurements at a comparable flow velocity. Furthermore, we found that higher EPS amount led to a higher apparent shear modulus of the biofilm at given flow velocity. The equilibrium shear modulus was also higher when the EPS ratio was relatively high. In general, the predicted equilibrium shear modulus of the biofilms was in the range of 3.5–5.7 Pa, which is consistent with experimental measurements of *P. aeruginosa* and *Staphylococcus aureus* reported in literature (Rupp et al., 2005; Stoodley et al., 1999). The nonlinear stress–strain characteristics of biofilms at large strains predicted by the simulations were comparable with some key experimental findings by rheometer measurements (Jana et al., 2020).

## AUTHOR CONTRIBUTIONS

Yuqing Xia, Pahala G. Jayathilake, Paul Stoodley, and Jinju Chen designed the research. Yuqing Xia performed the simulation work and acquired the data. Yuqing Xia and Jinju Chen did data analysis. Pahala G. Jayathilake, and Bowen Li contributed to data analysis. Yuqing Xia, Pahala G. Jayathilake, and Jinju Chen prepared the original draft. Jinju Chen and Pahala G. Jayathilake provided the overall guidance of the work. All the authors contributed to the writing of the manuscript, revised it, and approved the final version.

## CONFLICT OF INTEREST

The authors declare no conflict of interest.

## Supporting information

Supporting information.

## Data Availability

The authors confirm that the data supporting the findings of this study are available within the article and its supplementary materials.

## References

[bit28146-bib-0001] Abe, Y. , Skali‐Lami, S. , Block, J.‐C. , & Francius, G. (2012). Cohesiveness and hydrodynamic properties of young drinking water biofilms. Water Research, 46(4), 1155–1166. 22221338 10.1016/j.watres.2011.12.013

[bit28146-bib-0002] Ali, A. , Johnson, J. A. , Franco, A. A. , Metzger, D. J. , Connell, T. D. , Morris, J. G. , & Sozhamannan, S. (2000). Mutations in the extracellular protein secretion pathway genes (EPS) interfere with rugose polysaccharide production in and motility of Vibrio cholerae. Infection and Immunity, 68(4), 1967–1974. 10722590 10.1128/iai.68.4.1967-1974.2000 PMC97374

[bit28146-bib-0003] Antunes, J. , Leão, P. , & Vasconcelos, V. (2019). Marine biofilms: Diversity of communities and of chemical cues. Environmental Microbiology Reports, 11(3), 287–305. 10.1111/1758-2229.12694 30246474

[bit28146-bib-0004] Banerjee, D. , Shivapriya, P. , Gautam, P. K. , Misra, K. , Sahoo, A. K. , & Samanta, S. K. (2020). A review on basic biology of bacterial biofilm infections and their treatments by nanotechnology‐based approaches. Proceedings of the National Academy of Sciences, India, Section B: Biological Sciences, 90(2) 243–259.

[bit28146-bib-0005] Böl, M. , Ehret, A. E. , Bolea Albero, A. , Hellriegel, J. , & Krull, R. (2013). Recent advances in mechanical characterisation of biofilm and their significance for material modelling. Critical Reviews in Biotechnology, 33(2), 145–171. 22642670 10.3109/07388551.2012.679250

[bit28146-bib-0006] Bos, R. , Van der Mei, H. C. , & Busscher, H. J. (1999). Physico‐chemistry of initial microbial adhesive interactions—Its mechanisms and methods for study. FEMS Microbiology Reviews, 23(2), 179–230. 10234844 10.1111/j.1574-6976.1999.tb00396.x

[bit28146-bib-0007] Brindle, E. R. , Miller, D. A. , & Stewart, P. S. (2011). Hydrodynamic deformation and removal of *Staphylococcus epidermidis* biofilms treated with urea, chlorhexidine, iron chloride, or DispersinB. Biotechnology and Bioengineering, 108(12), 2968–2977. 21732324 10.1002/bit.23245

[bit28146-bib-0008] Capdeville, B. , & Rols, J. (1992). Introduction to biofilms in water and wastewater treatment. Biofilms—Science and Technology (pp. 13–20). Springer.

[bit28146-bib-0009] Capurro, M. , & Barberis, F. (2014). 9 ‐ Evaluating the mechanical properties of biomaterials. In P. Dubruel , & S. Van Vlierberghe (Eds.), Biomaterials for Bone Regeneration (pp. 270–323). Woodhead Publishing. 10.1533/9780857098104.2.270

[bit28146-bib-0010] Chen, J. , Bader, D. , Lee, D. , & Knight, M. (2011). Finite element modeling of cell deformation when chondrocyte seeded agarose is subjected to compression. In: *8th International Conference on Cell & Stem Cell Engineering (ICCE)*.

[bit28146-bib-0011] Chen, J. , & Lu, G. (2012). Finite element modelling of nanoindentation based methods for mechanical properties of cells. Journal of Biomechanics, 45(16), 2810–2816. 23017378 10.1016/j.jbiomech.2012.08.037

[bit28146-bib-0012] Costa, O. Y. , Raaijmakers, J. M. , & Kuramae, E. E. (2018). Microbial extracellular polymeric substances: Ecological function and impact on soil aggregation. Frontiers in Microbiology, 9, 1636. 30083145 10.3389/fmicb.2018.01636 PMC6064872

[bit28146-bib-0013] de Carvalho, C. C. (2018). Marine biofilms: A successful microbial strategy with economic implications. Frontiers in Marine Science, 5, 126.

[bit28146-bib-0014] Danese, P. N. , Pratt, L. A. , & Kolter, R. (2000). Exopolysaccharide production is required for development of *Escherichia coli* K‐12 biofilm architecture. Journal of Bacteriology, 182(12), 3593–3596. 10852895 10.1128/jb.182.12.3593-3596.2000 PMC101973

[bit28146-bib-0015] Davies, D. (2003). Understanding biofilm resistance to antibacterial agents. Nature Reviews Drug Discovery, 2(2), 114–122. 12563302 10.1038/nrd1008

[bit28146-bib-0016] Erskine, E. , MacPhee, C. E. , & Stanley‐Wall, N. R. (2018). Functional amyloid and other protein fibers in the biofilm matrix. Journal of Molecular Biology, 430(20), 3642–3656. 30098341 10.1016/j.jmb.2018.07.026 PMC6173796

[bit28146-bib-0017] Fang, H. H. , Chan, K.‐Y. , & Xu, L.‐C. (2000). Quantification of bacterial adhesion forces using atomic force microscopy (AFM). Journal of Microbiological Methods, 40(1), 89–97. 10739347 10.1016/s0167-7012(99)00137-2

[bit28146-bib-0018] Flemming, H.‐C. , Neu, T. R. , & Wozniak, D. J. (2007). The EPS matrix: The “house of biofilm cells”. Journal of Bacteriology, 189(22), 7945–7947. 17675377 10.1128/JB.00858-07 PMC2168682

[bit28146-bib-0019] Flemming, H.‐C. , & Wingender, J. (2010). The biofilm matrix. Nature Reviews Microbiology, 8(9), 623–633. 20676145 10.1038/nrmicro2415

[bit28146-bib-0020] Gloag, E. S. , Fabbri, S. , Wozniak, D. J. , & Stoodley, P. (2020). Biofilm mechanics: Implications in infection and survival. Biofilm, 2, 100017. 33447803 10.1016/j.bioflm.2019.100017 PMC7798440

[bit28146-bib-0021] Gloag, E. S. , Turnbull, L. , Huang, A. , Vallotton, P. , Wang, H. , Nolan, L. M. , Mililli, L. , Hunt, C. , Lu, J. , & Osvath, S. R. (2013). Self‐organization of bacterial biofilms is facilitated by extracellular DNA. Proceedings of the National Academy of Sciences of the United States of America, 110(28), 11541–11546. 23798445 10.1073/pnas.1218898110 PMC3710876

[bit28146-bib-0022] Greenshields, C. J. (2017). *OpenFoam user guide*. Version 6. OpenFOAM Foundation Ltd July.

[bit28146-bib-0023] Hibbing, M. E. , Fuqua, C. , Parsek, M. R. , & Peterson, S. B. (2010). Bacterial competition: Surviving and thriving in the microbial jungle. Nature Reviews Microbiology, 8(1), 15–25. 10.1038/nrmicro2259 19946288 PMC2879262

[bit28146-bib-0024] Houari, A. , Picard, J. , Habarou, H. , Galas, L. , Vaudry, H. , Heim, V. , & Di Martino, P. (2008). Rheology of biofilms formed at the surface of NF membranes in a drinking water production unit. Biofouling, 24(4), 235–240. 18392991 10.1080/08927010802023764

[bit28146-bib-0025] Israelachvili, J. N. (2011). Intermolecular and surface forces. Academic Press.

[bit28146-bib-0026] Jafari, M. , Desmond, P. , van Loosdrecht, M. C. , Derlon, N. , Morgenroth, E. , & Picioreanu, C. (2018). Effect of biofilm structural deformation on hydraulic resistance during ultrafiltration: A numerical and experimental study. Water Research, 145, 375–387. 30173098 10.1016/j.watres.2018.08.036

[bit28146-bib-0027] Jana, S. , Charlton, S. G. , Eland, L. E. , Burgess, J. G. , Wipat, A. , Curtis, T. P. , & Chen, J. (2020). Nonlinear rheological characteristics of single species bacterial biofilms. NPJ Biofilms and Microbiomes, 6(1), 1–11. 32286319 10.1038/s41522-020-0126-1 PMC7156450

[bit28146-bib-0028] Jayathilake, P. G. , Gupta, P. , Li, B. , Madsen, C. , Oyebamiji, O. , González‐Cabaleiro, R. , Rushton, S. , Bridgens, B. , Swailes, D. , & Allen, B. (2017). A mechanistic Individual‐based Model of microbial communities. PLoS One, 12(8), e0181965. 28771505 10.1371/journal.pone.0181965 PMC5542553

[bit28146-bib-0030] Klapper, I. , Rupp, C. J. , Cargo, R. , Purvedorj, B. , & Stoodley, P. (2002). Viscoelastic fluid description of bacterial biofilm material properties. Biotechnology and Bioengineering, 80(3), 289–296. 12226861 10.1002/bit.10376

[bit28146-bib-0031] Li, B. , Taniguchi, D. , Gedara, J. P. , Gogulancea, V. , Gonzalez‐Cabaleiro, R. , Chen, J. , McGough, A. S. , Ofiteru, I. D. , Curtis, T. P. , & Zuliani, P. (2019). NUFEB: A massively parallel simulator for individual‐based modelling of microbial communities. PLoS Computational Biology, 15(12), e1007125. 31830032 10.1371/journal.pcbi.1007125 PMC6932830

[bit28146-bib-0032] Lower, S. K. (2005). Directed natural forces of affinity between a bacterium and mineral. American Journal of Science, 305(6‐8), 752–765.

[bit28146-bib-0033] Peterson, B. W. , Busscher, H. J. , Sharma, P. K. , & Van Der Mei, H. C. (2014). Visualization of microbiological processes underlying stress relaxation in Pseudomonas aeruginosa biofilms. Microscopy and Microanalysis, 20(3), 912–915. 24621783 10.1017/S1431927614000361

[bit28146-bib-0034] Plimpton, S. (1995). Fast parallel algorithms for short‐range molecular dynamics. Journal of Computational Physics, 117(1), 1–19.

[bit28146-bib-0035] Rupp, C. J. , Fux, C. A. , & Stoodley, P. (2005). Viscoelasticity of *Staphylococcus aureus* biofilms in response to fluid shear allows resistance to detachment and facilitates rolling migration. Applied and Environmental Microbiology, 71(4), 2175–2178. 15812054 10.1128/AEM.71.4.2175-2178.2005 PMC1082509

[bit28146-bib-0036] Safari, A. , Tukovic, Z. , Walter, M. , Casey, E. , & Ivankovic, A. (2015). Mechanical properties of a mature biofilm from a wastewater system: From microscale to macroscale level. Biofouling, 31(8), 651–664. 26371590 10.1080/08927014.2015.1075981

[bit28146-bib-0037] Sehar, S. , & Naz, I. (2016). Role of the biofilms in wastewater treatment. *Microbial biofilms‐importance and applications*, 121–144.

[bit28146-bib-0038] Shaw, T. , Winston, M. , Rupp, C. J. , Klapper, I. , & Stoodley, P. (2004). Commonality of elastic relaxation times in biofilms. Physical Review Letters, 93(9), 098102. 15447143 10.1103/PhysRevLett.93.098102

[bit28146-bib-0039] Singh, R. , Paul, D. , & Jain, R. K. (2006). Biofilms: Implications in bioremediation. Trends in Microbiology, 14(9), 389–397. 16857359 10.1016/j.tim.2006.07.001

[bit28146-bib-0040] Song, X. , Xiong, Z. , Kong, L. , Wang, G. , & Ai, L. (2018). Relationship between putative eps genes and production of exopolysaccharide in *Lactobacillus casei* LC2W. Frontiers in Microbiology, 9, 1882. 30174661 10.3389/fmicb.2018.01882 PMC6107683

[bit28146-bib-0041] Stoodley, P. , Cargo, R. , Rupp, C. J. , Wilson, S. , & Klapper, I. (2002). Biofilm material properties as related to shear‐induced deformation and detachment phenomena. Journal of Industrial Microbiology and Biotechnology, 29(6), 361–367. 12483479 10.1038/sj.jim.7000282

[bit28146-bib-0042] Stoodley, P. , Lewandowski, Z. , Boyle, J. D. , & Lappin‐Scott, H. M. (1999). Structural deformation of bacterial biofilms caused by short‐term fluctuations in fluid shear: An in situ investigation of biofilm rheology. Biotechnology and Bioengineering, 65(1), 83–92. 10440674

[bit28146-bib-0043] Sun, R. , Xiao, H. , & Sun, H. (2018). Investigating the settling dynamics of cohesive silt particles with particle‐resolving simulations. Advances in Water Resources, 111, 406–422.

[bit28146-bib-0044] Syamlal, M. , Rogers, W. , O'Brien, T. , & Documentation, M. (1993). *Theory guide*. US Department of Energy, Morgantown, WV.

[bit28146-bib-0045] Tasneem, U. , Yasin, N. , Nisa, I. , Shah, F. , Rasheed, U. , Momin, F. , Zaman, S. , & Qasim, M. (2018). Biofilm producing bacteria: A serious threat to public health in developing countries. Journal of Food Sciences and Nutrition, 1(2), 25–31.

[bit28146-bib-0046] Thompson, A. P. , Plimpton, S. J. , & Mattson, W. (2009). General formulation of pressure and stress tensor for arbitrary many‐body interaction potentials under periodic boundary conditions. The Journal of Chemical Physics, 131(15), 154107. 20568847 10.1063/1.3245303

[bit28146-bib-0047] Van Rijn, L. C (1984). Sediment transport, part I: Bed load transport. Journal of Hydraulic Engineering, 110(10), 1431–1456.

[bit28146-bib-0048] Vinogradov, A. , Winston, M. , Rupp, C. J. , & Stoodley, P. (2004). Rheology of biofilms formed from the dental plaque pathogen Streptococcus mutans. Biofilms, 1(1), 49–56.

[bit28146-bib-0049] Wloka, M. , Rehage, H. , Flemming, H.‐C. , & Wingender, J. (2004). Rheological properties of viscoelastic biofilm extracellular polymeric substances and comparison to the behavior of calcium alginate gels. Colloid and Polymer Science, 282(10), 1067–1076.

[bit28146-bib-0050] Xia, Y. , Jayathilake, P. G. , Li, B. , Zuliani, P. , & Chen, J. (2021). CFD–DEM modelling of biofilm streamer oscillations and their cohesive failure in fluid flow. Biotechnology and Bioengineering, 118(2), 918–929. 33146404 10.1002/bit.27619

[bit28146-bib-0051] Yadav, S. K. , & Sanyal, S. (2019). Biofilms: The good and the bad. Biofilms in Human Diseases: Treatment and Control (pp. 13–26). Springer.

[bit28146-bib-0052] Zhu, H. , Zhou, Z. , Yang, R. , & Yu, A. (2007). Discrete particle simulation of particulate systems: Theoretical developments. Chemical Engineering Science, 62(13), 3378–3396.

