# Supplementary material for: Coupled CFD‐DEM modeling to predict how EPS affects bacterial biofilm deformation, recovery and detachment under flow conditions
Source: Biotechnol Bioeng. 2022 Jun 2;119(9):2551–63. doi: 10.1002/bit.28146 (PMC9544383; doi:10.1002/bit.28146)
Supplement: Supplementary file 1 — Supporting information. [file BIT-119-2551-s001.docx]

Coupled CFD-DEM modelling to predict how EPS affects bacterial biofilm deformation, recovery and detachment under flow conditions

Yuqing Xia^1^, Pahala Gedara Jayathilake^2^, Bowen Li^3^, Paolo Zuliani^3^, David Deehan^4,5^, Jennifer Longyear ^6^, Paul Stoodley^7,8^, Jinju Chen^1,^ *

^1^ School of Engineering, Newcastle University, Newcastle upon Tyne, NE1 7RU, U.K.

^2^ Department of Oncology, University of Oxford, Oxford, OX3 7DQ, U.K.

^3^ School of Computing, Newcastle University, Newcastle upon Tyne, NE1 7RU, U.K.

^4^ The Medical School, Newcastle University, Newcastle upon Tyne, NE1 7RU, U.K.

^5^ Freeman Hospital, Orthopaedics department. Newcastle upon Tyne, NE7 7DN, U.K.

^6^ Marin, Protective, and Yacht Coatings, AkzoNobel, Gateshead, NE10 0JY, U.K.

^7^ Department of Microbial Infection and Immunity and the Department of Orthopaedics, The Ohio State University, Columbus, OH, 43210, USA.

^8^National Centre for Advanced Tribology at Southampton (nCATS), National Biofilm Innovation Centre (NBIC), Department of Mechanical Engineering, University of Southampton, Southampton, S017 1BJ, UK.

* Correspondence: jinju.chen@newcastle.ac.uk; Tel.: +44-191-208-5434

Table S1 Kinetic parameters for biofilm growth.

| Parameter | Symbol | Value | Unit | Reference |
| --- | --- | --- | --- | --- |
| Simulation domains dimension | $L_{x}$, $L_{y}$, $L_{z}$ | 300, 40, 100 | $\mu$m | Chosen |
| Grid dimensions | $N_{x}$, $N_{y}$, $N_{z}$ | 90, 12, 30 | - | Chosen |
| Diffusion coefficient for substrate | $D_{s}$ | 1.6E-9 | m^2^ s^-1^ | (Jayathilake et al., 2017) |
| Nutrient concentrations | $S_{S}$ | 1.0E-4 | kg m^-3^ | Chosen |
| Kinetics and yields | | | |  |
| Maximum specific growth rate | $\mu_{m,HET}$ | 2.8E-4 | s^-1^ | (Jayathilake et al., 2017) |
| Substrate affinity | $K_{S}$ | 3.5E-5 | kg m^-3^ | (Jayathilake et al., 2017) |
| Yield coefficient | $Y_{HET}$ | 6.1E-1 | g COD_EPS_/  g COD_S_ | (Ni et al., 2009) |

Table S2 The dimension of the preformed biofilm. The EPS amount within the biofilm was controlled by the EPS formation coefficient. There was no significant difference among average biofilm height, roughness and porosity for biofilms with different EPS. The error bars were standard deviation based on three replicates.

| EPS growth Yield | 0.12 | 0.14 | 0.16 | 0.18 | 0.20 |
| --- | --- | --- | --- | --- | --- |
| EPS volume ratio | 20 % | 32 % | 40 % | 46 % | 51 % |
| Average biofilm height (µm) | 17.8 ± 0.5 | 19.6 ± 0.1 | 24.0 ± 0.8 | 25.0 ± 0.9 | 28.5 ± 0.3 |
| Max. biofilm height (µm) | 31.2 ± 3.0 | 33.8 ± 1.2 | 38.3 ± 1.2 | 40.3 ± 3.7 | 42.7 ± 3.2 |
| Biofilm RMS roughness (µm) | 5.7 ± 0.8 | 6.1 ± 0.3 | 6.2 ± 0.7 | 6.7 ± 0.9 | 7.0 ± 1.3 |
| Porosity of the biofilms (%) | 83.0 ± 1.6 | 81.4 ± 0.6 | 81.1 ± 0.6 | 79.6 ± 1.8 | 78.3 ± 1.6 |

Biofilm surface roughness is calculated as the root mean square (RMS) roughness by the following formula (Whitehouse, 2002):

roughness = ${(\frac{1}{L_{x}L_{y}}\iint{(h\left( x,y \right)-\bar{h})}^{2}dxdy)}^{1/2}$ , (S1)

where $h\left( x,y \right)$ is the height of the biofilm in z direction at the location $\left( x,y \right)$ on the substratum, $\bar{h}$ is the average height of the biofilm:

$\bar{h}$ = $\frac{1}{L_{x}L_{y}}\iint h(x,y)dxdy$ , (S2)

The porosity of the preformed biofilm is simply calculated as:

$porosity= \frac{volume of voids}{total volume}\times100\%$ (S3)

where the total volume is computed by multiplying the bottom area and the maximum height of biofilm, the voids volume equals the total volume minus the volume of biofilm.


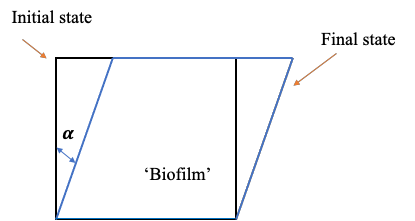


Figure S1. Schematic diagram illustrating biofilm deformation and engineering shear strain.

Figure S2. The measurement of engineering shear strain of the deformed biofilm by the flow.


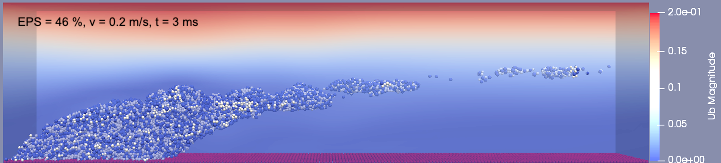


Figure S3. Biofilm deformation at inlet flow velocity of 0.2 m/s, t = 3 ms.


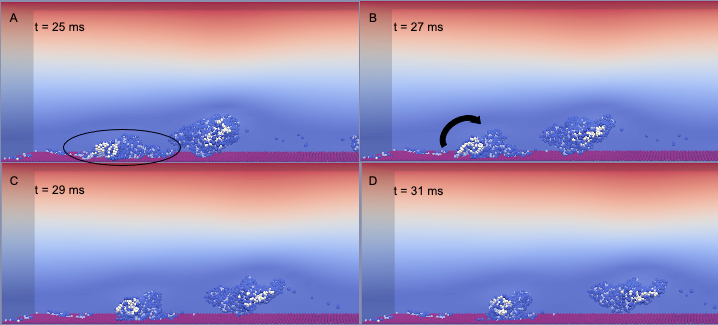


Figure S4. (A) - (D) The rolling motion of biofilm (with 46 % EPS) observed at different timepoints in simulation, inlet fluid velocity is 0.3 m/s.


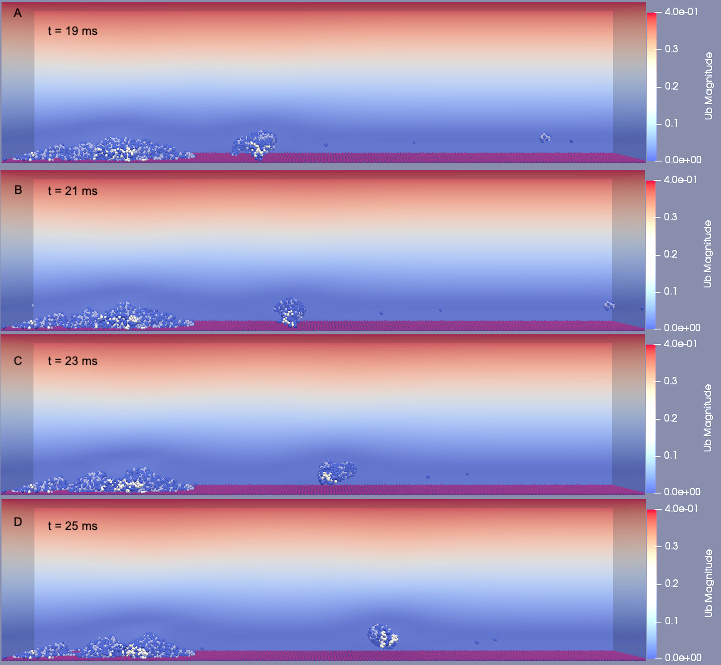


Figure S5. (A) - (D) The rolling motion of biofilm (with 46 % EPS) observed at different timepoints in simulation, inlet fluid velocity is 0.4 m/s.


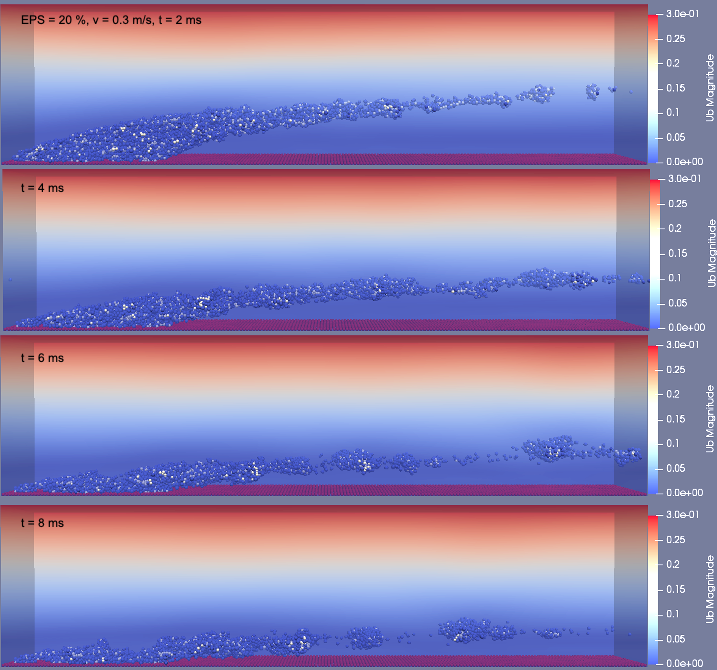


Figure S6. The deformation and detachment of biofilm (with 20 % EPS) during initial 8 ms, the inlet fluid velocity here is 0.3 m/s.


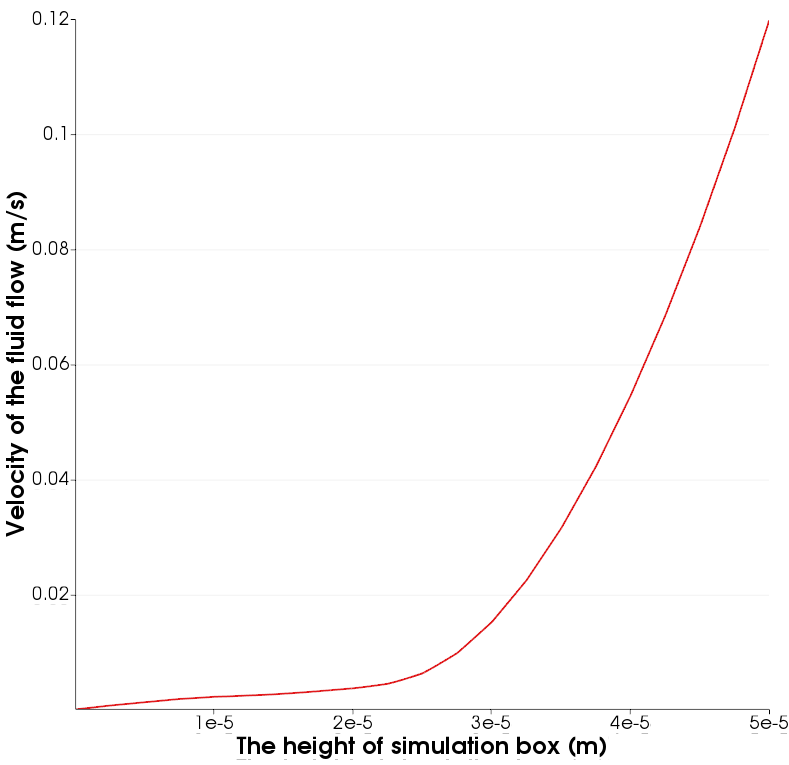


Figure S7. Inlet fluid velocity varied with the height of simulation box since the fluid flow was applied along the top wall, Therefore, the biofilms with different height would be subjected to different fluid shear force.

**Experimental measurements**

The experiments for biofilm deformation were carried out in the microfluidic chambers fabricated as mentioned above. *Bacillus subtilis* stock was thawed and 10 µl was added to 20 ml Tris (Tris (hydroxymethyl) aminomethane) and placed in a shaker overnight to prepare the liquid culture.

The cultures were injected into the channels using a syringe pump at 35 µl/h. The flow rate was maintained for 2 h to allow bacterial cells to attach to the walls of the chambers. The culture solution was removed and culture broth was injected into the system. The flow was maintained at 35 µl/h for 36-48 h to allow biofilm formation. Once the biofilm is formed, the pump is run at higher flow rate (500 µl/h) to remove any loosely attached clumps of biofilm from the channel. 1 µm polystyrene beads were added to the culture broth which makes it convenient to use Particle Image Velocimetry (PIV) to track particle motion in order to calculate the velocity field. Biofilm experiments are carried out by ramping the flow rate to the maximum flow rate of 25000 µl/h and the biofilm surface compression was detected by a high-speed camera (Photron Fastcam mini UX) at 8000 fps. Then, the normal stress acting on biofilms were calculated based on the change of velocity field on biofilm surfaces using an open-source code MATLAB code known as Queen 2 (Dabiri et al., 2014). The normal stress was determined by the relative deformation of biofilm.

Figure S8 displays a representative image of tracking the normal deformation of biofilms (y direction) and stress-strain curve at a max flow rate of 25000 µl/h (0.14 m/s). The apparent Young’s modulus was 18.7 ± 0.2 Pa. If assuming the Poisson’s ratio is about 0.49, the apparent shear modulus will be about 6.3Pa which is comparable to the biofilm shear modulus predicted in our simulations.

Figure S8. A representative (A) image of tracking the normal deformation of biofilms (y direction) and (B) stress-strain curve at a max flow rate of 25000 µl/h (0.14 m/s for the given flow cell).

Dabiri, J. O., Bose, S., Gemmell, B. J., Colin, S. P., & Costello, J. H. (2014). An algorithm to estimate unsteady and quasi-steady pressure fields from velocity field measurements. *Journal of Experimental Biology, 217*(3), 331-336. <https://doi.org/10.1242/jeb.092767>

Jayathilake, P. G., Gupta, P., Li, B., Madsen, C., Oyebamiji, O., González-Cabaleiro, R., Rushton, S., Bridgens, B., Swailes, D., Allen, B., McGough, A. S., Zuliani, P., Ofiteru, I. D., Wilkinson, D., Chen, J., & Curtis, T. (2017). A mechanistic Individual-based Model of microbial communities. *PLoS One, 12*(8), e0181965. <https://doi.org/10.1371/journal.pone.0181965>

Ni, B. J., Fang, F., Xie, W. M., Sun, M., Sheng, G. P., Li, W. H., & Yu, H. Q. (2009, Mar). Characterization of extracellular polymeric substances produced by mixed microorganisms in activated sludge with gel-permeating chromatography, excitation-emission matrix fluorescence spectroscopy measurement and kinetic modeling. *Water Res, 43*(5), 1350-1358. <https://doi.org/10.1016/j.watres.2008.12.004>

Whitehouse, D. (2002). 3 - Profile and areal (3D) parameter characterization. In D. Whitehouse (Ed.), *Surfaces and Their Measurement* (pp. 48-95). Kogan Page Science. <https://doi.org/https://doi.org/10.1016/B978-190399601-0/50003-7>
